# Education differences in sickness absence and the role of health behaviors: a prospective twin study

**DOI:** 10.1186/s12889-020-09741-y

**Published:** 2020-11-11

**Authors:** K. B. Seglem, R. Ørstavik, F. A. Torvik, E. Røysamb, M. Vollrath

**Affiliations:** 1 Department of Mental Disorders, Norwegian Institute of Public Health, P. O. Box 222, Skøyen, 0213 Oslo, Norway; 2 Department of Mental Health and Suicide, Norwegian Institute of Public Health, P. O. Box 222, Skøyen, 0213 Oslo, Norway; 3 Centre for Fertility and Health, Norwegian Institute of Public Health, P. O. Box 222, Skøyen, 0213 Oslo, Norway; 4 Department of Psychology, University of Oslo, P. O. Box 1094, Blindern, 0317 Oslo, Norway; 5 Department of Child Health, Norwegian Institute of Public Health, P. O. Box 222, Skøyen, 0213 Oslo, Norway; 6 PROMENTA Research Center, Department of Psychology, University of Oslo, P. O. Box 1094, Blindern, 0317 Oslo, Norway

**Keywords:** Sickness absence, Education, Health behaviors, Causality, Twin study, Familial confounding, Genes, Norway

## Abstract

**Background:**

Long-term sickness absences burden the economy in many industrialized countries. Both educational attainment and health behaviors are well-known predictors of sickness absence. It remains, however, unclear whether these associations are causal or due to confounding factors. The co-twin control method allows examining causal hypotheses by controlling for familial confounding (shared genes and environment). In this study, we applied this design to study the role of education and health behaviors in sickness absence, taking sex and cohort differences into account.

**Methods:**

Participants were two cohorts of in total 8806 Norwegian twins born 1948 to 1960 (older cohort, mean age at questionnaire = 40.3, 55.8% women), and 1967 to 1979 (younger cohort, mean age at questionnaire = 25.6, 58.9% women). Both cohorts had reported their health behaviors (smoking, physical activity and body mass index (BMI)) through a questionnaire during the 1990s. Data on the twins’ educational attainment and long-term sickness absences between 2000 and 2014 were retrieved from Norwegian national registries. Random (individual-level) and fixed (within-twin pair) effects regression models were used to measure the associations between educational attainment, health behaviours and sickness absence and to test the effects of possible familial confounding.

**Results:**

Low education and poor health behaviors were associated with a higher proportion of sickness absence at the individual level. There were stronger effects of health behaviors on sickness absence in women, and in the older cohort, whereas the effect of educational attainment was similar across sex and cohorts. After adjustment for unobserved familial factors (genetic and environmental factors shared by twin pairs), the associations were strongly attenuated and non-significant, with the exception of health behaviors and sickness absence among men in the older cohort.

**Conclusions:**

The associations between educational attainment, health behaviors, and sickness absence seem to be confounded by unobserved familial factors shared by co-twins. However, the association between health behaviors and sickness absence was consistent with a causal effect among men in the older cohort. Future studies should consider familial confounding, as well as sex and age/cohort differences, when assessing associations between education, health behaviors and sickness absence.

**Supplementary Information:**

**Supplementary information** accompanies this paper at 10.1186/s12889-020-09741-y.

## Background

High levels of sickness absence are a growing concern in many industrialized countries. Norway has one of the highest sickness absence rates with approximately 6% of working days lost over the past decade [1]. Sickness absence increases considerably across age, and women have a higher level than men [2]. For an individual, staying away from work when ill is often necessary to ensure good health. Long term sickness absence can, however, also have a negative impact on a person’s health, and is a risk factor for permanent disability and lifelong exclusion from the labor market [3, 4]. Despite sickness absence being more than a measure of morbidity, e.g. influenced by the nature of one’s work [5, 6] and socio-political structures [7], there is a clear education gradient in sickness absence that parallels the well-known education gradient in health. Individuals with lower educational attainment, a key dimension of socioeconomic status, are at higher risk of sickness absence and labor market exclusion [8]. Studies of education and sickness absence borrow largely from theoretical perspectives on the widely studied “education-health gradient” [9], thus positing that educational attainment has a causal effect on sickness absence [10, 11]. An important mechanism, partly explaining education differences in health, is differences in health behaviors [9, 12]. Knowledge about the influence of health behaviors on sickness absence is limited [13–19], but lifestyle or health behaviors have been documented as one explanation for socioeconomic differences in sickness absence [6, 10, 11, 20, 21]. The etiological processes underlying the association between education, health behaviors and sickness absence is poorly understood, but results from some studies indicate that these associations may be confounded by unobserved familial factors, i.e. genetic and/or environmental factors shared by co-twins [22, 23].

Education is considered as an important individual determinant of later medically confirmed sickness absence [22, 24, 25]. Individuals with higher educational level have lower levels of sickness absence than those with lower educational level, indicating better health and worklife functioning [5]. Education is typically completed by early adulthood, while other indicators of socioeconomic status, such as occupational class and income, are determined later [26, 27]. Compared to income, educational attainment is a stronger socio-economic determinant of sickness absence in societies where differences in income levels are relatively low, such as in Nordic countries [22, 24], which is why we focus on education in the present study. Furthermore, education differs from other socio-economic indicators in that it primarily indicates differences in non-material resources such as general knowledge, and health literacy, which maylead to healthier behaviors [9]. The importance of lifestyle or health behaviors for sickness absence has been studied to a limited degree only. Much of the evidence focuses on single health behaviors, is based on relatively small sample sizes and findings have been mixed [13–18]. However, a large observational study of cohorts from France, Finland, and the UK found that lifestyle-related factors including BMI, physical activity, smoking and alcohol consumption were all associated with sickness absence [19]. Two previous studies have investigated whether lifestyle or health behaviors explain educational differences in sickness absence [10, 11]. A population-based study among 30–64 year olds in Finland, found that lifestyle factors including smoking, physical exercise, sleeping problems, alcohol consumption and obesity altoghether explained about 15% of the educational differences in sickness absence, with a stronger effect among women [10]. A study of workers in six companies in the Netherlands, found that overweight/obesity explained 21% of educational differences, after working conditions and perceived general health was accounted for [11]. Together, these studies indicate that lifestyle-related factors play a role in the mechanisms through which education affects sickness absence. However, these studies were observational, thus, inferences about causality could not be made. There is increasing appreciation that health behaviors do not co-occur within individuals by chance, but that they tend to cluster. Those who smoke cigarettes are more likely to drink excessive amounts of alcohol and less likely to eat healthy and be physically active [28–31]. Poor health behaviors are also more prevalent among individuals with less education [9]. Instead of targeting specific health behaviors, some argue that multiple behaviors need to be targeted, in order for interventions to have an effect on health [23, 32]. A previous randomized trial showed that an intervention involving physical exercise, health advice and smoking cessation had an effect on sickness absence [33]. Other intervention studies limited to physical exercise [34] and overweight [35] alone, did not appear to have any effects. Based on the evident clustering of health behaviors and that the sum of several health behaviors seems more important for sickness absence than a particular health behavior, we use a health behavior index in the present study to focus on broad explanations for the role of health behaviors.

Recently, a growing body of studies using causal inference designs failed to fully support the hypothesis that socio-economic status exerts a causal effect on health [36–39]. The co-twin control method represents one such design, where the aim is to mimic a counterfactual situation: Monozygotic (MZ) twin pairs are genetically identical while dizygotic (DZ) pairs share on average 50% of their genes, just like other siblings. If raised together, both share their family environment. In the co-twin control method, the size of associations between exposure and outcome is compared with the corresponding within MZ (and DZ) associations. For example, if educational attainment statistically predicts sickness absence in the population, and we find a similar effect among MZ-twins with different levels of educational attainment (within pair analyses), this supports that educational attainment is causally related to sickness absence. If, on the other hand, we observe that the population-based association disappear in the within pair analyses, the initial association is probably due to confounding by unmeasured confounding by genes or shared environmental factors. Subgroup analyses within MZ and DZ twins pairs allow to differentiate between confounding due to genes or shared environment.

A previous twin study of young Norwegian adults based partly on the same data as the present study [22] showed that within DZ twins, the effect of education on sickness absence was attenuated. Within MZ twins, who share both the family environment and all of their genes, the effect of education on sickness absence was negligible and reduced to non-significance, indicating that mainly genetic influences explained the association between education and sickness absence in young adulthood. In an older sample of middle-aged Swedish twins, Samuelsson and colleagues [40] found that the association between education and disability pensioning, a construct strongly related to sickness absence, was also confounded by familial factors. In contrast, a twin study of health behaviors and risk for disability pensioning found an effect independent of familial factors [23].

### The present study

In this paper, we aim to add to the existing literature on educational and health behavior differences in sickness absence, by employing a co-twin control design. Based on previous findings [10, 11], we hypothesized that educational attainment and health behaviors are independent predictors of sickness absence, and that health behaviors partly explain educational differences in sickness absence. We further hypothesized, based on previous twin studies [22, 23, 40], that the association between education and sickness absence would not be consistent with a causal explanation, but that the association between health behaviors and sickness absence would. Due to well-known sex and age differences in level of sickness absence, but limited knowledge of causal factors underlying these differences [2], we will explore the effects by age/birth cohort and sex subgroups.

## Methods

### Particpants and design

Information from three Norwegian registries was linked using national identity numbers. The first was the Norwegian twin registry, comprising information on 40,639 twins born between 1895 and 1960 and between 1967 and 1991, respectively. For the present study, we selected two cohorts of twins. The older cohort was born between 1948 and 1960 and had completed a health questionnaire between 1990 and 1999 (median = 1995). The younger cohort of twins was born between 1967 and 1979 and had completed a similar health questionnaire between 1998 and 1999. The mean age of the two cohorts when answering the questionnaires was 40.3 years and 25.6 years, respectively. The second registry (the Historical-Event Database) contained information on each twin’s sickness absence and employment. The third registry contained information about each twin’s highest completed education (the Norwegian Educational Database). Sickness absences were retrieved for the period 2000 to 2014, ensuring that the twins had completed the health questionnaires before the first recorded sickness absence. We excluded 348 participants who had fewer than 100 working days registered throughout the 15 year follow-up period. For the older cohort, only same-sex twins were available. Among the final sample of 8806 twins, there were 2755 complete pairs (480 monozygotic (MZ) male, 389 dizygotic (DZ) male, 777 MZ female, 635 DZ female, and 474 unlike-sex twin pairs) and 3296 single twins. Questionnaire items and genotyping of a subsample determined zygosity [41].

In this longitudinal, population-based twin study, we employed a co-twin control design. The basics of this design is explained in the introduction. The Regional Committee for Medical and Health Research Ethics (case 2015/405) approved of the study.

### Measures

#### Sickness absence

We computed *sickness absence* taking the ratio of sickness absence days to contracted working days, ranging from 0 to 100%. The mandatory Norwegian Insurance Scheme covers sickness absences exceeding 16 days and up 365 days during a calendar year. We excluded sickness absences granted for problems or illnesses related to “pregnancy, childbearing, family planning” [42] since those were relevant for women in the younger cohort only.

#### Educational attainment

Data on *educational attainment* was available annually from 1980 to 2014 from the Norwegian Educational Database administered by Statistics Norway. Here the Norwegian Standard Classification of Education [43] distinguished eight levels, ranging from “no education” to “Ph.D. or equivalent”. We simplified this classification by merging technical diplomas with undergraduate levels and Ph.D.s with master degrees, resulting in five educational levels. To ensure completeness, we used data from 2014 when the youngest participants were 35 years old.

#### Health behaviors

Health-related lifestyle factors were based on self-reported information. *Leisure-time physical activity* (“How often do you exercise?”) included categories of never, less than once a week, one to two times per week and three times per week or more. Categories were reverse coded prior to analyses, so that higher score reflects less physical activity. *Body mass index (BMI)* was used as a proxy indicator for diet or overeating [32, 44]. BMI was calculated based on weight (“How much do you weigh?”) and height (“How tall are you?”). We first categorized BMI as underweight (lower than 18.5 kg/m^2^), normal weight (18.5–24.9 kg/m^2^), overweight (25.0–29.9 kg/m^2^), and obesity (30.0 kg/m^2^ or higher). Due to few individuals in the underweight and obese categories and a u-shaped association with sickness absence, we dichotomized BMI into normal weight (0) versus not (1). *Smoking* was assessed with the questions “Do you currently smoke?” and “If you quit smoking, how old were you then?”. We categorized smoking status as current or past smoker (1) and non-smoker (0). The internal-consistency reliability for the three health behavior variables was low as expected, i.e. KR-20 = .26.

A *health behavior* composite score was computed using principal component analysis (PCA) of the three health behavior measures, and saving the factor scores (i.e., standardized, weighted sum score). The Kaiser-Meyer-Olkin (KMO) measure verified the sampling adequacy for the analysis, KMO = .52, and all KMO values for individual items were > .51 which is above the acceptable limit of .50 [45]. Bartlett’s test of sphericity χ^2^ (3) = 434.12, *p* = < .001, indicated that correlations between items were sufficiently large for PCA. The items clustered on one component with an eigenvalue over Kaiser’s criterion of 1 and explained 41.57% of the variance. Factor loadings were .40 for BMI, .76 for physical activity and .72 for smoking. Higher score reflects less healthy behaviors, i.e. an unhealthy lifestyle.

#### Sex and birth cohort

*Sex* referred to that which was assigned at birth (men = 1, women = 2), and was together with *cohort* (birth year) available from the Norwegian Twin Registry.

### Statistical analyses

Models included observations with complete information on all model variables. Number of missing cases are reported in Table 1. We performed the analyses using STATA SE version 15 [46].

**Table 1:** Descriptive statistics by cohort and sex

	Cohort 1948–60		Cohort 1967–79	
	Men	Women	Men	Women
	*N* = 1437	*N* = 1815	*N* = 2280	*N* = 3274
**Age at health study, M (SD)**	41.0 (3.7)	39.7 (4.0)	25.7 (3.7)	25.5 (3.7)
**Ages at SA follow-up, range**	40–66	40–66	21–47	21–47
**Educational attainment**				
1 Primary/lower secondary	13.9%	19.2%	6.6%	6.8%
2 Upper secondary, basic	21.1%	25.6%	3.5%	4.0%
3 Upper secondary, final year	26.4%	16.9%	31.5%	27.2%
4 Post-secondary/ Undergrad.	25.8%	33.1%	37.6%	46.3%
5 Master or higher	12.9%	5.3%	20.8%	15.8%
Missing n	3	6	1	2
**BMI**				
Normal (18.5–25 kg/m^2^)	59.3%	78.6%	71.5%	78.2%
Missing n	25	69	72	141
**Physical activity**				
Never	42.0%	42.6%	4.9%	3.0%
< once a week	15.8%	13.8%	27.7%	25.0%
1–2 times per week	26.5%	29.3%	36.8%	40.3%
3 = < times per week	15.7%	14.3%	30.6%	31.6%
Missing n	41	79	25	27
**Smoking**				
Current/Past	61.6%	60.8%	41.9%	46.5%
Missing n	151	168	21	32
**Health behavior composite (z-score)**^**a**^	.55 (1.05)	.41 (0.95)	−.23 (0.93)	−.27 (0.89)
Missing n	187	267	111	190
**Proportion of sickness absence**				
M (SD)	6.3% (11.1)	9.3% (13.4)	2.9% (6.3)	5.8% (8.7)^b^

^a^Health behavior composite contains BMI, physical activity and smoking; ^b^Does not include sickness absence granted for pregnancy related diagnoses. Including this would yield a mean of 7.4% (SD: 9.2)

Careful inspection of scatterplots showed that education, health behaviors and sickness absence were linearly related. In the first set of analyses, the associations of education and health behaviors with sickness absencewere assessed with random-effects generalized least squares (GLS) regression using the twins as individuals. Standard errors and CIs were adjusted for dependence between twins in pairs using robust variances (Stata command *xtreg*, option *re*). We first estimated a model including the effects of education, sex and cohort on sickness absence, then added the effect of the health behavior composite. Sex and cohort differences were examined using two- and three-way interaction terms. We finally calculated to what extent the association between education and sickness absence were reduced when health behaviors were included in the regression equation.

Secondly, we repeated the analyses using within twin pair models, by running fixed-effects models separately for monozygotic and dizygotic twins (Stata command *xtreg*, option f*e*). This approach separates the effects of familial and genetic confounding, respectively. An attenuation of estimates in DZ twin pairs would indicate familial (genes and or shared environment) confounding while further attenuation in MZ twin pairs would suggest genetic confounding [47]. Models were run for the full sample and subgroups of sex and cohorts .

## Results

### Descriptives

Table 1 provides descriptive statistics for each cohort and sex. In the older and younger.

cohorts, 38.5% versus 60.5% had higher education (beyond upper secondary),, t (8792) = − 28.93, *p <* .001. Men scored higher on the health behavior composite, indicating more unhealthy behaviors, than women in both the older, t (2732) = 3.81, *p <* .001, and in younger cohort, t (5159) = 2.97, *p* = .003. The older cohort scored higher on unhealthy behaviors t (7893) = 33.38, *p <* .001. The overall incidence of any sickness absence was 70.2%, i.e. a majority of participants were granted sickness absence during the years 2000 to 2014. A total of 6.4% of all working days between 2000 and 2014 were lost to sickness absence. There was a lower sickness absence proportion among men (6.3% in the older cohort and 2.9% in the younger cohort, t (3640) = 11.88, *p <* .001) than among women (9.3% in the older cohort and 5.8% in the younger cohort, t (4995) = 10.78, *p <* .001).

**Fig. 1:**
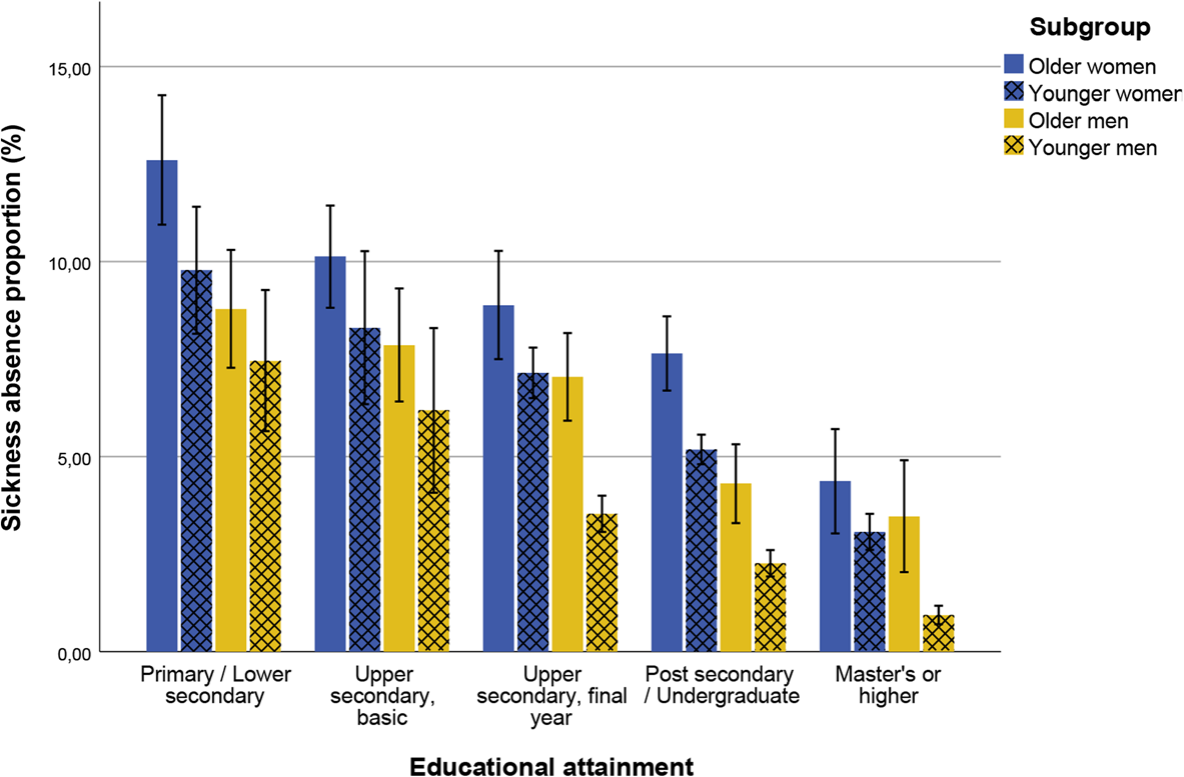
Education level and sickness absence by subgroup. Whiskers represent 95% confidence intervals

Figure 1 shows a bar graph of educational attainment differences in annual mean sickness absence proportion in the follow-up years from 2000 to 2014 among women and men in the older and younger cohort. In the total sample, the difference in sickness absence varied from 10.3% among those with lowest education (primary/lower secondary) to 2.4% among those with the highest level (Master’s degree or higher). Despite differing levels of sickness absence, there was a clear negative relationship with educational attainment in all subgroups.

### Individual-level analyses

Table 2 shows the results of the random-effect models predicting proportion of sickness absence from educational attainment and health behaviors for the total sample, including tests of interaction with sex and cohort. Educational attainment was standardized to be directly comparable to health behaviors. The first model shows the association between education and sickness absence adjusted for birth cohort and sex, and accounting for twin dependency. The regression coefficient indicates that as educational attainment increased by one standard deviation (SD), the mean annual sickness absence proportion decreased with 1.83 percentage points. In unstandardized units, and more comparable to the raw data in Fig. 1, the coefficient was − 1.57 (95% CI: − 1.75, − 1.38), indicating that with each increasing level in educational attainment, sickness absence decreased with 1.57 percentage points. This means that based on the general sickness absence proportion of 6.4%, one SD increase in education reduces sickness absence by 29%, while each increase in education level reduces sickness absence with 25%. The between R-squared for Model 1 indicated that 10% of the individual variation in sickness absence was explained.

**Table 2:** Random-effects generalized least squares (GLS) regression model with sickness absence regressed on standardized predictors

	Model 1	Model 2	Model 3	Model 4	Model 5	Model 6
Education	−1.83*** (− 2.04, − 1.61)	−1.58*** (− 1.81, − 1.35)	−1.47*** (− 1.81, − 1.14)	−1.63*** (− 1.97, − 1.29)	−1.58*** (− 1.81, − 1.35)	−1.59*** (− 1.82, − 1.36)
Sex (female)	2.81*** (2.38, 3.23)	2.93*** (2.50, 3.37)	2.94*** (2.50, 3.38)	2.93*** (2.49, 3.37)	2.93*** (2.49, 3.36)	2.94*** (2.50, 3.38)
Cohort (younger)	−2.41*** (− 2.89, −1.94)	−1.83*** (− 2.34, − 1.33)	−1.82*** (− 2.33, − 1.32)	−1.82*** (− 2.33, − 1.31)	−1.84*** (− 2.34, − 1.33)	−1.73*** (− 2.24, − 1.21)
Health behaviors		0.97*** (0.74, 1.20)	0.98*** (0.75, 1.21)	0.97*** (0.74, 1.20)	0.69*** (0.37, 1.02)	1.28*** (0.92, 1.64)
Sex*education			−0.19 (− 0.62, 0.24)	–	–	–
Cohort*Education				0.10 (−0.36, 0.55)	–	–
Sex*Health behaviors					0.51* (0.10, 0.93)	–
Cohort*Health behaviors						−0.51* (−0.97, − 0.05)
N	8794	8039	8039	8039	8039	8039

Model 1: Education, sex, cohort, and accounting for twin dependency; Model 2: Model 1 + health behavior composite; Model 3: Model 2 + interaction term with sex and education; Model 4: Model 2 + interaction term with cohort and education; Model 5: Model 2 + interaction term with sex and health behaviors; Model 6: Model 2 + interaction term with cohort and health behaviors

95% confidence intervals in parantheses; * *P* < 0.05; ** *P* < 0.01; *** *P* < 0.001

In the second model we added the health behavior composite. This resulted in a 14% reduction in the education-sickness absence coefficient, indicating a small degree of overlap and potential mediation. Yet, both education and health behaviors showed unique statistically significant contributions. Based on the general sickness absence proportion of 6.4%, one SD increase in unhealthy behaviors was prospectively associated with 0.97 percentage points or a 15% increase in sickness absence. The between R-squared for the model was .11, indicating that the composite of health behaviors only explained an additional 1% of the individual variation in sickness absence. Models 3 and 4 include two-way interactions to investigate whether there were statistically significant sex and cohort effects in the education gradient in sickness absence. Results indicated no interaction effects. Model 5 shows a stronger effect of health behaviors on sickness absence among women than men, and model 6 a weaker effect of health behaviors in the younger than the older cohort. There were no statistically significant three-way interactions.

To better understand how education and health behaviors are associated and since health behaviors are generally regarded as mediators of the effect of education on sickness absence, we ran an additional model predicting health behaviors from educational attainment for the whole sample, adjusting for birth year and sex, and accounting for twin dependency (not shown in table). Next, we tested sex and cohort differences in effects of education on health behaviors with two- and three-way interaction terms. Results from the adjusted model show that individuals with higher education scored more favourable on the health behavior composite (β = − 0.24, 95% CI = − 0.26, − 0.22, *p* < .001), women scored more favourable than men (β = − 0.10, 95% CI = − 0.14, − 0.06, *p* < .001) and the younger cohort more favourable than the older cohort (β = − 0.58, 95% CI = − 0.63, − 0.53, *p* < .001). Furthermore, the effect of education on health behaviors showed statistically significant interactions with sex and cohort: The effect of education was stronger in women than in men (β = 0.08, 95% CI = 0.04, 0.12, *p* < .001), and stronger in the older than the younger cohort (β = − 0.11, 95% CI = − 0.15, − 0.06, *p* < .001).

### Within-twin pair analyses

Table 3 shows the associations between education, health behaviors, and sickness absence within DZ and MZ twin pairs for the total sample. In DZ pairs, the association between education and sickness absence remained, i.e. higher education was associated with lower sickness absence. This association was slightly attenuated when adding health behaviors in Model 2, but health behaviors did not show a statistically significant association with sickness absence. Within MZ pairs, the association of both education and health behaviors with sickness absence was small and not statistically significant.

**Table 3:** Within-twin pair associations between education, health behaviors and sickness absence in the total sample

	Model 1		Model 2	
	DZ	MZ	DZ	MZ
Education^a^	−1.40** (− 2.22, − 0.58)	− 0.07 (− 0.80, 0.67)	−1.25** (− 2.10, − 0.41)	−0.14 (− 0.94, 0.66)
Health behaviors^a^			0.63 (−0.15, 1.40)	0.36 (−0.36, 1.09)
N pairs	1020	1256	857	1082

Model 1: Education; Model 2: Model 1 + health behavior composite; ** *P* < 0.01; Coefficients are reported with 95% confidence intervals

^a^Education and health behaviors were standardized prior to model entry

**Fig. 2:**
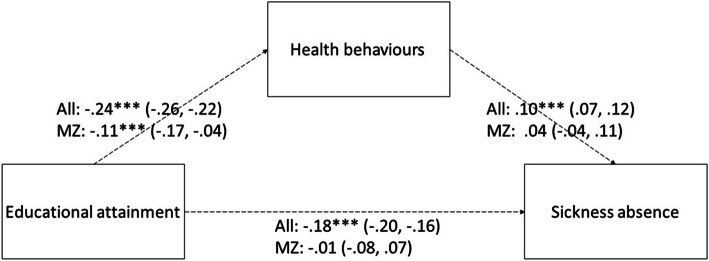
All standardized coefficients from regression models with total sample adjusted for sex, cohort and twin dependency (first line) and within MZ twins (second line). Coefficients for sickness absence regressed on health behaviors (higher score indicates less healthy behaviors) was additionally adjusted for educational attainment

Figure 2 shows the standardized regression coefficients for the associations between all main variables for the total sample and within MZ pairs (full adjustment of familial confounders) presented as a mediation model. This shows that the association of health behaviors and educational attainment with sickness absence was confounded by familial (shared environmental and/or genetic) factors. The association between educational attainment and health behaviors, on the other hand, was attenuated, yet remained statistically significant after control for familial factors.

Next, we ran fixed-effect models for each sex and cohort group separately (See Figures S1 a-d in the online supplementary material). Fixed-effects models were run with DZ and MZ twin pairs combined to increase statistical power when running analyses in subgroups. Results were similar as for the fixed-effect model in the total sample, but with some exceptions. The most notable and robust difference was found between the cohorts in the association between educational attainment and health behaviors. In the older cohort the education-health behaviors association almost disappeared after adjusting for familial factors. In contrast, the education-health behaviors association in the younger cohort was somewhat attenuated and remained statistically significant (Women: β = −.10, *p* = .011, Men: β = −.18, *p* < .001). We checked the robustness of this association by running analyses within MZ twins only, confirming the results (Women: β = −.14, 95% CI = − 0.25, − 0.03, *p* = .014; Men: β = −.13, 95% CI = − 0.24, − 0.02, *p* = .025). Due to the similar results for women and men in the younger cohort, we combined the sexes to increase statistical power. In the younger cohort, the association between education and health behaviors was similar in MZ and DZ twins (β = −.13, 95% CI = − 0.21, − 0.06, *p* = .001 and β = −.13, 95% CI = − 0.22, − 0.04, *p* = .007, respectively), indicating partial confounding by mainly shared environmental factors. Another exception was the association between health behaviors and sickness absence, which among men in the older cohort was enhanced and statistically significant in the within MZ twin analyses (β = .26, 95% CI = 0.00, 0.52, *p* = .048).

### Robustness analyses

To validate our findings, we performed several robustness checks. We reran the analyses with years of education (M = 14.32, SD = 2.74, range = 7–22) obtained from national registry data, yielding essentially the same results as with five educational levels (see Tables S1 and S2 in the online supplementary material). Second, we computed separate analyses for those who had completed their highest education *before* participating in the health study, to ensure temporal alignment. The analyses yielded essentially the same results (see Tables S3 and S4). Since more participants in the younger cohort had not completed their highest level of education before participating in the health study (51.3%), we ran additional models to check whether this affected the results in the younger cohort (Figures S2 a and b). No substantial differences were found.

## Discussion

The key findings of the present study were that on the population level, educational attainment and health behaviors were prospectively associated with sickness absence among both women and men, as well as older and younger cohorts. Controlling for genetic and shared environmental factors, however, showed that these associations appeared to be confounded by familial factors and were therefore probably not causal. One exception was the association between health behaviors and sickness absence among men in the older cohort. These findings are an important contribution to the sickness absence literature, suggesting that a larger focus on the role of genetic mechanisms is warranted. The main findings are subsequently discussed.

The findings that low educational attainment and poor health behaviors were associated with higher levels of sickness absence replicates previous observational studies (e.g. 19, 24). In addition to both education and health behaviors exerting main effects on sickness absence, there was also a degree of overlap between them. As previously shown in other studies [10, 11], we found that the effect of education on sickness absence was reduced once health behaviors were controlled for. This is in line with theories of health behaviors being mediators in the education-health outcome link [48, 49]. However, our analyses of within-twin pair differences might give reason to reconsider these interrelationships.

Adjusting for factors shared by co-twins reduced the associations between education and sickness absence and between health behaviors and sickness absence (except for men in the older cohort). The reduction of the education-sickness absence association in the younger cohort sample has previously been documented [22]. Our study confirms these findings for an even longer follow-up time of 6 years, until the year 2014, when the younger cohort have reached the ages 35–47, as well as extending these findings to hold also for the older cohort with follow-up until retirement age. We are not aware of any other previous twin studies that investigated whether the interrelationships between education, health behaviors and sickness absence are consistent with causal hypotheses. However, our results are consistent with a previous twin study of disability pensioning in Sweden, showing that the association between education and disability pensioning is confounded by familial factors [40]. Another Swedish twin study showed that the association between a combination score of health behaviors and disability pensioning was unclear [23]. This could reflect that Ropponen and Svedberg combined alcohol consumption, which they found to have a protective effect, together with tobacco use and low physical activity, found to be risk factors. Previous studies have found a U-shaped association between alcohol use and sickness absence, with abstainers and high level users having more sickness absence [50, 51]. At the same time, alcohol use shows a more complex and heterogeneous pattern of association with socio-economic status (SES) than many other public health challenges, with higher SES often being associated with higher alcohol consumption [52]. Different ways of operationalizing and combining health behaviors make comparisons across studies complicated. Nevertheless, knowledge of how various health behaviors interact in different groups or contexts is important for researchers and policy makers in the hope of improving health-related behaviors and reduce sickness absence in the population.

In the present study, results indicated a causal link between health behaviors and sickness absence among men in the older cohort. In the younger cohort, health behaviors may not have had enough time to exert an effect on health or sickness absence. Why health behaviors did not seem to have a causal link to sickness absence among women in the older cohort, however, is more difficult to explain. One suggestion is that there may be selection effects, as the older cohort belongs to a generation where women typically stayed more at home. Therefore it may have been easier for these women than the men to reduce their participation in or exit the labour force if experiencing health problems. However, women in the older cohort had higher levels of sickness absence, indicating that such selection effects were not overriding. Another observation is that men in the older cohort showed more unfavourable health behaviours as measured by the composite and higher BMI in particular. This could indicate that sickness absence due to lifestyle diseases may be more prevalent among older men than women. This is consistent with previous observational studies showing that obesity is particularly associated with sickness absence due to digestive and circulatory diseases [19], and that several health behaviors, including smoking and BMI, has shown stronger associations with medically confirmed sickness absence among men than women [53].

We also examined whether the association between education and health behaviors was consistent with a causal explanation. Interestingly, the association between educational attainment and health behaviors was only partly confounded and remained statistically significant after familial control in the younger cohort, but not the older. This shows that education and health behaviors have become more causally related in younger cohorts. This corresponds to previous studies in the US showing that health behaviors exert a stronger impact on the education gap in mortality at younger than older ages [54], and that risky health behaviors have become more concentrated among more recent cohorts of individuals with lower education [55]. This could be due to improved quality of education, more health campaigns and interventions from health authorities, or it could be due to sociocultural mechanisms leading to clustering of better or worse health behaviors in the upper and lower end of socioeconomic positions. The latter explanation also fits with the increasing socioeconomic segregation seen in populations of many industrialized countries [56].

### Strengths and limitations

The strengths of this study include the prospective study design, the long follow-up period, the fact that we relied on high-quality registry data regarding exposure and outcome, and the genetically informative design that captured population data covering the entire age span of the Norwegian working population. Furthermore, in the present study, persons with at least 100 employment days, and regardless of the hours of employment, during the 15-year follow-up period were included. With these wide inclusion criteria, we are likely to include persons who only work part-time due to health reasons and persons who fall out of the labour market for various reasons. By including all persons who are eligible for sickness absence benefits some time during follow-up, we include a broader segment of the population, which we believe make the findings more generalizable with regard to sickness absence in the population.

The limitations of the study are first, that despite being able to use twin pairs as optimal matching of cases and controls, not all putative factors could be controlled for in this study. While the within-pair estimates are free from confounding from genetic and shared environmental facors, these estimates may be biased by non-shared confounders [57]. For example, health problems early in life in one twin may explain why this twin has lower education as well as poorer health behaviors such as lower physical activity. Second, sickness absence is a complex construct and our study has taken into account only some influential factors. Risk factors specific to work, family situation attributable to the person or work-home interference, as well as psychological trait factors may be important and should be considered when interpreting our findings. Third, due to restrictions in data accessibility we were only able to follow the twins until 2015. There has (except from the current situation with covid-19) however, been no major changes in patterns of sickness absence or levels of employment since. Fourth, we only had available information on long term sickness absence, i.e. at least 16 days. We therefore do not know if the same results apply for short term sickness absence. Finally, the results may not be generalizable to all settings, and are best generalizable to Nordic and European countries with similar welfare schemes, attitudes and cultures of health behaviors.

## Conclusions

To conclude, both educational attainment and health behaviors were independently associated with level of sickness absence, but these associations were strongly confounded by familial factors. Based on these findings, interventions aiming to increase educational attainment or improve overall health behaviors, despite their potential importance in improving public health, might not be the best strategy to reduce the rate of sickness absence. Future studies investigating education and health behaviors as predictors of sickness absence need to take familial confounding into account, as well as consider variations between sex and age/cohort groups.

## Supplementary Information

**Additional file 1.**

## Data Availability

The raw data is confidential and cannot readily be shared. Data may be shared with researchers obtaining permissions from The Norwegian Twin Registry, Statistics Norway, and the Regional Committees for Medical and Health Research Ethics.

## Abbreviations

MZ: Monozygotic
DZ: Dizygotic
BMI: Body mass index
PCA: Principal component analysis
KMO: Kaiser-Meyer-Olkin
GLS: Generalized least squares
SD: Standard deviation
SES: Socio-economic status
