# Supplementary material for: Education differences in sickness absence and the role of health behaviors: a prospective twin study
Source: BMC Public Health. 2020 Nov 11;20:1689. doi: 10.1186/s12889-020-09741-y (PMC7656504; doi:10.1186/s12889-020-09741-y)
Supplement: Supplementary file 1 — Additional file 1. [file 12889_2020_9741_MOESM1_ESM.zip › Supplementary materialR2.docx]

**Supplementary material**

**Table S1** Random-effects generalized least squares (GLS) regression model with sickness absence regressed on standardized predictors^1^

|  | Model 1 | Model 2 | Model 3 | Model 4 | Model 5 | Model 6 |
| --- | --- | --- | --- | --- | --- | --- |
| Education (yrs) | -1.71***  (-1.92, -1.49) | -1.44***  (-1.67, -1.22) | -1.22***  (-1.55, -0.89) | -1.66***  (-2.01, -1.30) | -1.39***  (-1.62, -1.16) | -1.39***  (-1.62, -1.16) |
| Sex (female) | 2.92***  (2.50, 3.35) | 3.03***  (2.60, 3.47) | 3.04***  (2.61, 3.48) | 3.02***  (2.59, 3.46) | 3.06***  (2.63, 3.50) | 3.08***  (2.64, 3.51) |
| Cohort (younger) | -2.53***  (-3.00, -2.06) | -1.92***  (-2.42, -1.41) | -1.90***  (-2.40, -1.39) | -1.86***  (-2.37, -1.35) | 1.38*  (0.17, 2.59) | 1.57*  (0.35, 2.79) |
| Health behaviors |  | 1.00***  (0.77, 1.23) | 1.01***  (0.78, 1.24) | 1.01***  (0.78, 1.24) | 0.65***  (0.33, 0.97) | 1.30***  (0.95, 1.66) |
| Sex*education |  |  | -0.40  (-0.82, 0.03) | - | - | - |
| Cohort*Education |  |  |  | 0.36  (-0.10, 0.81) | - | - |
| Sex*Health beh. |  |  |  |  | 0.56**  (0.15, 0.98) | - |
| Cohort*Health beh. |  |  |  |  |  | -0.58*  (-1.03, -0.12) |
| N observations | 8758 | 8005 | 8005 | 8005 | 8005 | 8005 |

Model 1: Education, sex, cohort, and accounting for twin dependency; Model 2: Model 1 + health behavior composite; Model 3: Model 2 + interaction term with sex and education; Model 4: Model 2 + interaction term with cohort and education; Model 5: Model 2 + interaction term with sex and health behaviors; Model 6: Model 2 + interaction term with cohort and health behaviors.

95% confidence intervals in parantheses; * *P* < 0.05; ** *P* < 0.01; *** *P* < 0.001

^1^ Education measured as number of years

**Table S2** Within-twin pair associations between education^1^, health behaviors and sickness absence in the total sample

|  | Model 1 | | Model 2 | |
| --- | --- | --- | --- | --- |
|  | DZ | MZ | DZ | MZ |
| Education (yrs) | -1.54***  (-2.36, -0.72) | 0.13  (-0.61, 0.87) | -1.33**  (-2.18, -0.49) | 0.04  (-0.75, 0.84) |
| Health behaviors |  |  | 0.58  (-0.19, 1.36) | 0.37  (-0.35, 1.10) |
| N pairs | 1012 | 1247 | 992 | 1231 |

Model 1: Education; Model 2: Model 1 + health behavior composite

95% confidence intervals in parantheses; *** *P* < 0.001

^1^Education measured as number of years

**Table S3** Random-effects generalized least squares (GLS) regression model with sickness absence regressed on standardized predictors^1^

|  | Model 1 | Model 2 | Model 3 | Model 4 | Model 5 | Model 6 |
| --- | --- | --- | --- | --- | --- | --- |
| Educational attainment | -1.92***  (-2.21, -1.63) | -1.64***  (-1.94, -1.33) | -1.63***  (-2.08, -1.19) | -1.60***  (-2.02, -1.18) | -1.64***  (-1.94, -1.33) | -1.66***  (-1.96, -1.35) |
| Sex (female) | 2.70***  (2.11, 3.30) | 2.80***  (2.19, 3.42) | 2.80***  (2.16, 3.44) | 2.81***  (2.19, 3.42) | 2.76***  (2.15, 3.38) | 2.80***  (2.18, 3.41) |
| Cohort (younger) | -2.48***  (-3.09, -1.86) | -2.01***  (-2.67, -1.35) | -2.01***  (-2.67, -1.35) | -2.03***  (-2.72, -1.35) | -2.01***  (-2.66, -1.35) | -1.88***  (-2.55, -1.22) |
| Health behaviors |  | 1.02***  (0.69, 1.34) | 1.02***  (0.69, 1.34) | 1.02***  (0.69, 1.34) | 0.88***  (0.44, 1.33) | 1.37***  (0.93, 1.82) |
| Sex*education |  |  | -0.01  (-0.59, 0.57) | - | - | - |
| Cohort*Education |  |  |  | -0.07  (-0.66, 0.52) | - | - |
| Sex*Health behaviors |  |  |  |  | 0.26  (-0.34, 0.86) | - |
| Cohort*Health behaviors |  |  |  |  |  | -0.73*  (-1.36, -0.10) |
| N observations | 5156 | 4692 | 4692 | 4692 | 4692 | 4692 |

Model 1: Education, sex, cohort, and accounting for twin dependency; Model 2: Model 1 + health behavior composite; Model 3: Model 2 + interaction term with sex and education; Model 4: Model 2 + interaction term with cohort and education; Model 5: Model 2 + interaction term with sex and health behaviors; Model 6: Model 2 + interaction term with cohort and health behaviors.

95% confidence intervals in parantheses; * *P* < 0.05; ** *P* < 0.01; *** *P* < 0.001

^1^ Run with subsample of those with completed education prior to measurement of health behaviors

**Table S4** Within-twin pair associations between education, health behaviors and sickness absence^1^

|  | Model 1 | | Model 2 | |
| --- | --- | --- | --- | --- |
|  | DZ | MZ | DZ | MZ |
| Educational attainment | -1.35  (-2.72, 0.02) | 0.75  (-0.66, 2.17) | -1.29  (-2.65, 0.08) | 0.76  (-0.66, 2.18) |
| Health behaviors |  |  | 1.14  (-0.16, 2.43) | 0.07  (-1.23, 1.36) |
| N pairs | 664 | 766 | 664 | 766 |

Model 1: Education; Model 2: Model 1 + health behavior composite

95% confidence intervals in parantheses

^1^ Run with subsample who had completed education prior to measurement of health behaviors

**Fig. S1 a-d** Standardized beta coefficients, with 95% confidence intervals, from regression models within each cohort and sex subgroup. Individual-level estimates adjusted for sex, birth year and twin dependency are shown in the first line. Within twin pair estimates (MZ/DZ twins together for higher statistical power when subgrouping) are shown in the second line. Within MZ twin pair estimates are shown in the third line. Sickness absence regressed on health behaviors was adjusted for educational attainment in all models. Subsample sizes were for women older cohort: All = 1809, MZ/DZ pairs = 539 and MZ pairs = 258; men older cohort: All = 1434, MZ/DZ pairs = 337 and MZ pairs = 155; women younger cohort: All = 3272, MZ/DZ pairs = 869 and MZ pairs = 519; men younger cohort: All = 2279, MZ/DZ pairs= 531 and MZ pairs = 325. * *P* < 0.05; ** *P* < 0.01; *** *P* < 0.001.

**Fig. S2 a and b** Standardized beta coefficients, with 95% confidence intervals, from regression models in women (n All = 1562; n MZ/DZ pairs = 258; n MZ pairs = 157) and men (n All = 1143; n MZ/DZ pairs = 162; n MZ pairs = 104) in the younger cohort who *had* *completed education prior to measurement of health behaviors*. Individual-level estimates adjusted for sex, birth year and twin dependency are shown in the first line. Within twin pair estimates (MZ/DZ twins together for higher statistical power when subgrouping) are shown in the second line. Within MZ twin pair estimates are shown in the third line. Sickness absence regressed on health behaviors was adjusted for educational attainment in both models. * *P* < 0.05; ** *P* < 0.01; *** *P* < 0.001.
