# Supplementary figures and images for: Education differences in sickness absence and the role of health behaviors: a prospective twin study
Source: BMC Public Health. 2020 Nov 11;20:1689. doi: 10.1186/s12889-020-09741-y (PMC7656504; doi:10.1186/s12889-020-09741-y)

**S1 a** Women older cohort

**
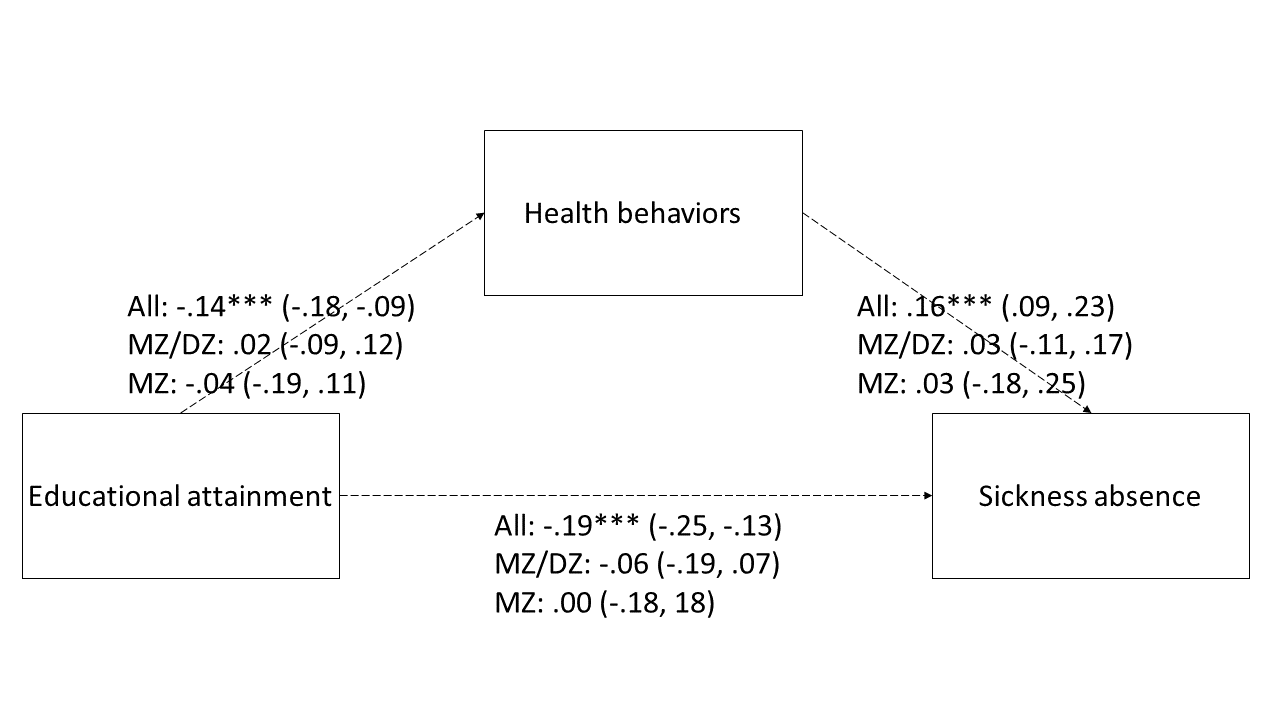
**

**S1 b** Men older cohort

**
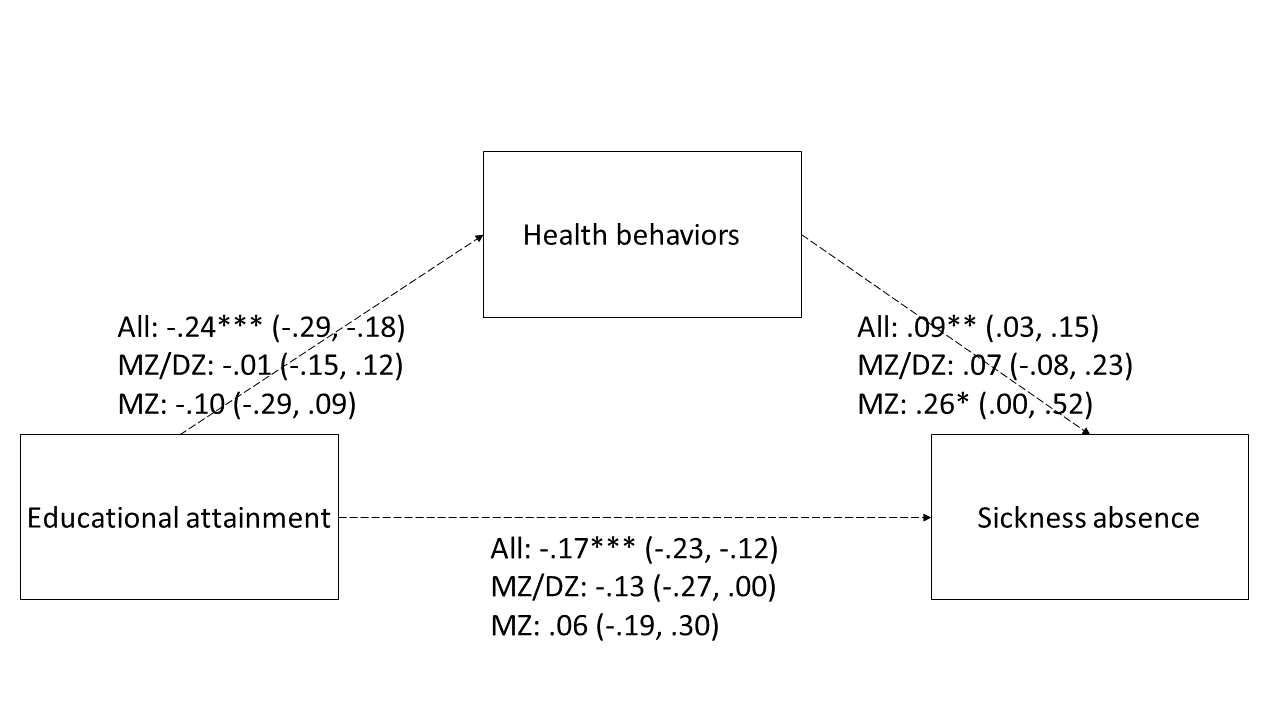
**

**S1 c** Women younger cohort

**
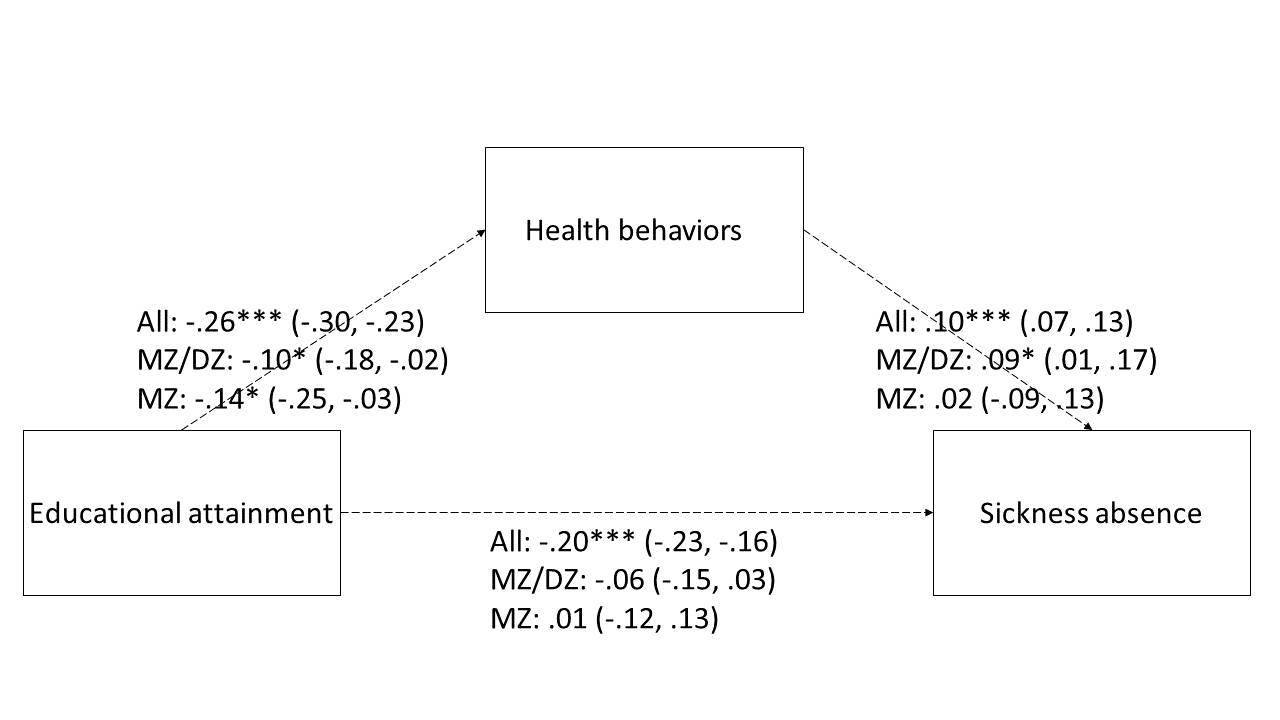
**

**S1 d** Men younger cohort

**
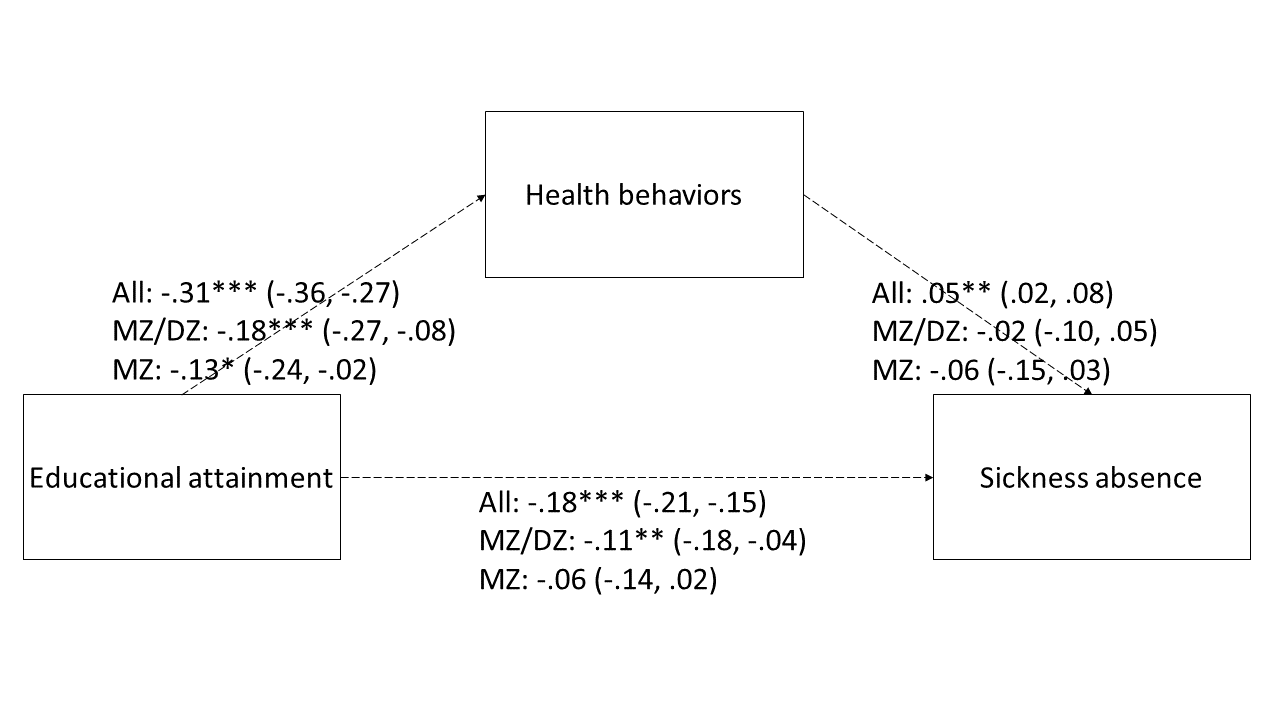
**

Supplement: Supplementary file 1 — Additional file 1. [file 12889_2020_9741_MOESM1_ESM.zip › Fig S1R2.docx]

**S2 a** Women younger cohort

**
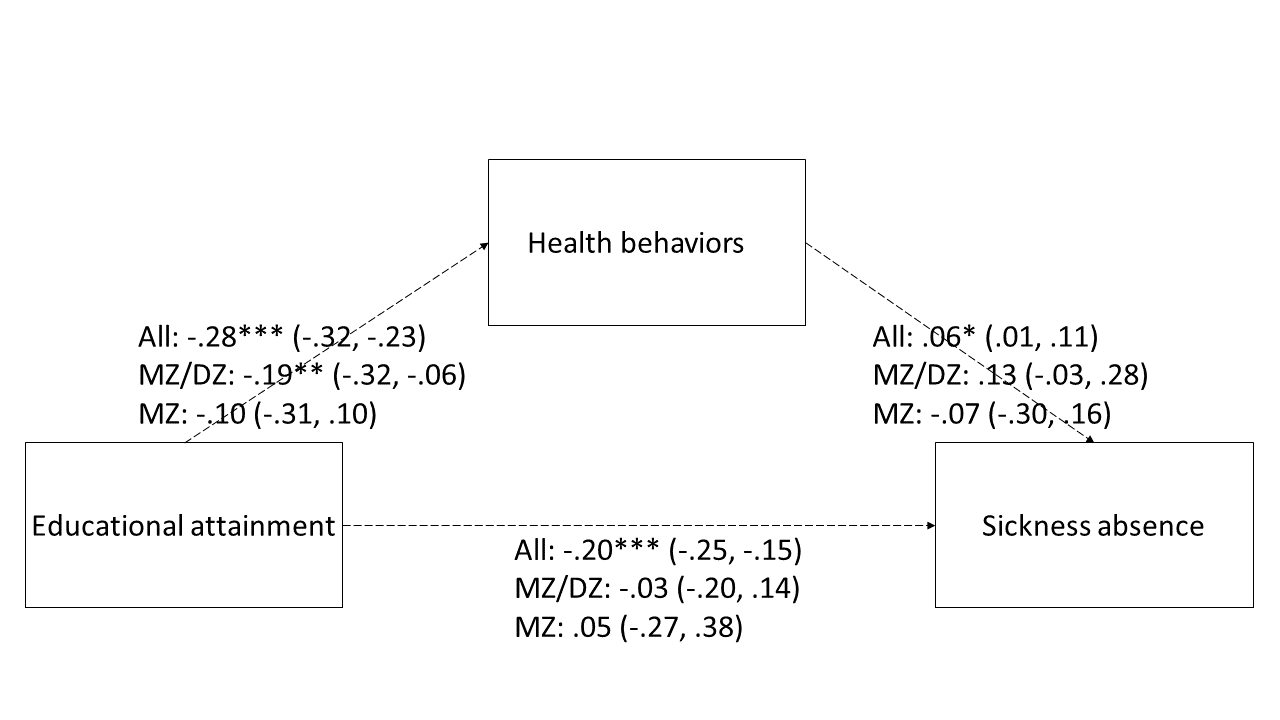
**

**S2 b** Men younger cohort**
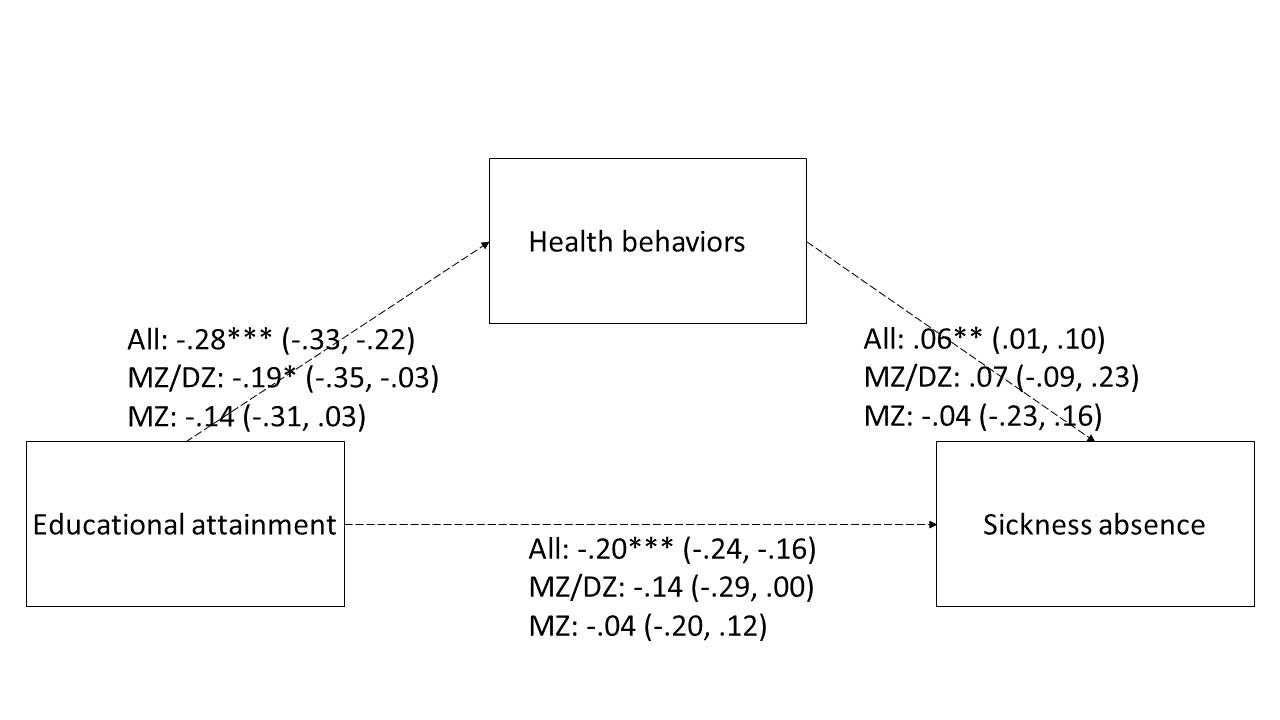
**

Supplement: Supplementary file 1 — Additional file 1. [file 12889_2020_9741_MOESM1_ESM.zip › Fig S2R2.docx]
